# Anti‐Human Endogenous Retrovirus and Anti‐Myelin Oligodendrocyte Glycoprotein Humoral Response in Cerebrospinal Fluid of Multiple Sclerosis Patients: A Case Control Study

**DOI:** 10.1111/1348-0421.70003

**Published:** 2025-07-22

**Authors:** Karin C. Garcia, Samuel N. Santos, Bruna R. Flose, Maria A. Juliano, Renan B. Domingues, Lucas M. Neves, Guilherme José Costa Silva, Liã B. Arruda, Augusto C. Penalva‐de‐Oliveira, Sandro L. de Andrade Matas, Marina T. Shio, Luiz H. da Silva Nali

**Affiliations:** ^1^ Pós Graduação em Ciências da Saúde Universidade Santo Amaro São Paulo Brazil; ^2^ Universidade Federal de São Paulo São Paulo Brazil; ^3^ Senne Liquor Diagnóstico São Paulo Brazil; ^4^ Pós Graduação em Saúde Única Universidade Santo Amaro São Paulo Brazil; ^5^ Wellcome Connecting Science Wellcome Genome Campus Hixton UK; ^6^ Departamento de Neurologia e Infectologia Instituto de Infectologia Emilio Ribas São Paulo Brazil; ^7^ Departamento de Neurologia Unifesp: setor Líquido cefalorraquidiano e Neuroinfectologia São Paulo Brazil

**Keywords:** cerebrospinal fluid, HERV‐K, HERV‐W, humoral response, multiple sclerosis, myelin oligodendrocyte glycoprotein

## Abstract

It is believed that human endogenous retrovirus (HERV)‐W plays a fundamental role in multiple sclerosis (MS) pathogenesis, probably by inducing humoral and cellular immunopathological response. HERVs present regions of similarity to myelin oligodendrocyte glycoprotein (MOG) and therefore could induce autoimmunity through the mechanism of molecular mimicry via cross‐humoral response. This study aimed to evaluate the impact of HERV‐mediated humoral response among MS patients by assessing IgG antibody levels of HERV‐W and K in cerebrospinal fluid (CSF). CSF samples were collected from MS patients (*n* = 25) and idiopathic intracranial hypertension (IIH) patients (*n* = 25). Serum samples from MS group (*n* = 25) were also analyzed. CSF samples were assessed for global and differential cell counts, analytical biochemistry, and oligoclonal bands analysis. ELISA was used to determine the serum and intrathecal (CSF) presence and concentration of anti‐MOG, anti‐HERV‐W‐env, and anti‐HERV‐K‐pol antibodies. ELISA findings revealed higher concentrations of anti‐MOG and HERV‐W IgG in MS compared to IIH (*p* < 0.01 and *p* = 0.0142 respectively), while HERV‐K IgG showed concentration to three HERV‐K peptides (*p* < 0.01). A positive correlation was also observed between serum and CSF antibody concentration for MOG (*r* = 0.47 *p* = 0.01), HERV‐W (*r* = 0.72 *p* < 0.01), and three HERV‐K peptides (*r* = 0.49, 0.57, 0.61 and *p* = 0.0126, *p* < 0.01, and *p* < 0.01, respectively) among MS patients. Our findings revealed high concentrations of anti‐HERV‐K and ‐W antibodies in serum and CSF among MS patients, suggesting a possible role of humoral immunopathological response. In addition, the positive correlation between serum and CSF antibody concentration indicates the potential application of serum levels of anti‐HERV and anti‐MOG as biomarkers for MS.

**Abbreviations**
CNS: central nervous system
CSF: cerebrospinal fluid
EDSS: expanded disability status scale
ELISA: enzyme‐linked immunosorbent assay
FBS: fetal bovine serum
HBTU: *N*,*N*′‐tetramethyl‐*O*‐benzotriazo‐1‐yluronium tetrafluoroborate
HERV: human endogenous retrovirus
HOBt: 1‐hydroxybenzotriazole
HPLC: high‐performance liquid chromatography
IgG: immunoglobulin G
IIH: idiopathic intracranial hypertension
MOG: myelin oligodendrocyte glycoprotein
MS: multiple sclerosis
OCB: oligoclonal band
PBS: phosphate buffer saline
RBC: red blood cells
SD: standard deviation
STROBE: strengthening the reporting of observational studies in epidemiology
UNISA: Universidade Santo Amaro

## 1 Introduction

Multiple sclerosis (MS) is a chronic autoimmune demyelinating disease of the central nervous system (CNS). It is one of the most common causes of neurological disability in young adults, affecting mainly women [1]. MS etiology is unknown; however, it is postulated to be triggered by an infectious agent, specially viruses. Among them, human endogenous retrovirus (HERV)‐W is the one with the most evidence of association with MS pathogenesis. Briefly, HERVs are ancient retroviruses that infected our ancestors' germline cells millions of years ago. They have been fixed into the human genomes and were transmitted through generations by retrotransposition, horizontal transmission, and later by Mendelian inheritance [2]. Nowadays, it is known that 8% of the human genome corresponds to HERV sequences [3].

The findings that have associated HERV‐W with the pathogenesis of MS include higher levels of HERV‐W expression and proviral load among MS patients [4, 5, 6], detection of HERV‐W env protein in active MS brain lesions [7, 8, 9], and induction of MS in animal model after exposition to HERV‐W‐env protein in mice [10].

MS patients usually present antibodies against myelin oligodendrocyte glycoprotein (MOG) [11, 12], a protein on the surface of oligodendrocytes. MOG‐immunoglobulin G (IgG) has been associated with demyelinating disease and rare cases of MS [13]. Interestingly, MOG shares similar regions with HERV transcripts, suggesting the potential of HERV to mediate an autoimmune response due to a possible molecular mimicry [14, 15]. Although the accumulated evidence pointing to a fundamental role of HERV‐W on MS pathogenesis, the specific mechanism for triggering autoimmune response through molecular mimicry is still poorly understood due to the lack of information on alternative self‐antigens sites for autoimmune response. Therefore, the aim of this study was to investigate the antibody response against HERV‐W, HERV‐K peptides, and MOG in the cerebrospinal fluid (CSF) of MS patients, to determine whether distinct HERVs peptides could induce different humoral response and to compare whether MS patients shows distinct humoral response than patients without neuroinflammatory diseases such as idiopathic intracranial hypertension (IIH).

## 2 Materials and Methods

### 2.1 Study Population

Our retrospective case‐control study has included CSF and serum samples from MS patients (*n* = 25) and CSF samples from patients who presented IIH (*n* = 25) as a control group, these individuals were selected as controls as they do not present inflammatory response on its pathogenesis [16].

Approximately 2 mL of CSF and 1.5 mL of serum were donated to the UNISA research laboratory and were kept at −80°C until use. Only samples collected within less than 6 months were included. Patients were classified into two groups as follows: CSF samples that had presented at least two oligoclonal bands (OCBs) were included in the MS group, and to compose the IIH only CSF samples who did not present any OCBs were included in this group. Importantly, samples to compose each group were conveniently collected, and the distribution of patients in both groups showed similar age and gender composition. Also, we have followed the strengthening the reporting of observational studies in epidemiology (STROBE) guideline for case control studies.

## 3 CSF Analysis

A global cell count was performed in Fuschs‐Rosenthal chamber and a neutrophil differential analysis was performed when the global count was greater than and equal to 4 cells/mm^3^. Neutrophils were identified with Leishman staining. CSF biochemical analytes (total protein, glucose, and lactate) were measured using the enzymatic colorimetric method. Albumin and IgG quantification were determined by nephelometry. The IgG index was provided by senne liquor lab and it was calculated as follows: (IgG [CSF] × albumin [serum]/IgG [serum] × albumin [CSF]) × 100. OCBs were detected through isoelectric focusing electrophoresis with hydragel CSF isofocusing electrophoresis kit (Sebia).

### 3.1 HERV‐W‐Env, HERV‐K‐Pol and MOG Peptides Synthesis

The peptides were synthesized in the biophysics lab of the Universidade Federal de São Paulo. Briefly, to infer the similarity between HERVs and MOG protein regions, publicly available sequences from HERV‐K pol (GenBank accession# M14123.1), HERV‐W Env (GenBank accession# AAI37382.1), and MOG (GenBank accession#: CAA52617) were assessed. Protein sequences were aligned and compared for similarity through SIM—Alignment Tool for Protein Sequences (https://web.expasy.org/sim/). We have selected regions with approximately 50% similarity to identify possible candidates for molecular mimicry. Eligible regions had at least five amino acids and part of the protein which were expressed outside of the membrane and were identified using TMpred Prediction of Transmembrane Regions and Orientation (https://embnet.vitalit.ch/software/TMPRED_form.html) [17]. Peptides were synthesized by solid‐phase 9‐fluorenylmethyloxycarbonyl on an automated multiple peptide synthesizer (PSSM‐8 system; Shimadzu, Tokyo, Japan) [18].

For the synthesis, the coupling reagent HBTU (*N*,*N*'‐tetramethyl‐*O*‐benzotriazo‐1‐yluronium tetrafluoroborate)/HOBt (1‐hydroxybenzotriazole) was used, in a NovaSyn TGR resin (Millipore, USA). Next, the resin was washed with TFA: thioanisole:1,2‐ethanedithiol:water (85:5:3:7) to remove the peptides, followed by a semi‐preparative HPLC purification step on a C18 column (Econosil Fisher Scientific, USA). The molecular weight and purity of the peptides (> 95%) were verified by reversed‐phase chromatography and mass spectrometry (Shimadzu, Japan).

### 3.2 Anti‐HERV‐W‐Env, Anti‐HERV‐K‐Pol and Anti‐MOG Antibodies Detection in CSF and Serum of MS Patients

The ELISA for MOG and HERVs was previously standardized [17]. Briefly, 96‐well plates (Nunc MaxiSorp, Thermo) were coated with 25 µL of each peptide (20 µg/mL) diluted in carbonate/bicarbonate buffer (pH 9.6) and incubated at 4°C for 24 h. Then, wells were blocked for 1 h with 3% fetal bovine serum (FBS) in wash buffer (PBS with 0.1% Tween‐20). Twenty‐five microliters of CSF samples diluted 1:2 in PBS or 25 µL of serum samples diluted in 1:500 was added to the wells for 1 h at 37°C. The plates were washed three times with wash buffer and incubated for 30 min at room temperature with peroxidase‐conjugated anti‐human IgG (Sigma) diluted in 0.1% PBS‐Tween and 1% FBS (1:5000). Subsequently, in each well it was added 50 µL of 1 mg/mL ABTS in 0.1M citrate buffer, pH 4.0 (Sigma) and hydrogen peroxide. The reaction was terminated with 1M NH4Cl. Plate reading (Loccus, LMR‐96) was performed at 405 nm.

### 3.3 Statistical Analysis

Data descriptive statistics and analyses were performed using GraphPad Prism version 6.0 (La Jolla, California). The normal test indicated that data distribution was normal, therefore, the *t*‐test was used to compare data from both MS and IIH groups. All tests were carried out assuming the probability of error (alpha) of 5%. Correlation analysis was performed using the Pearson test.

### 3.4 Ethics

Samples included in this study were collected and stored by Senne Liquor lab under the prescription of neurologists for neuro‐disorder diagnosis and were not obtained from this cohort for purpose of answering the objectives of this study. As these samples were designated for diagnosis, the leftover samples, that would be discarded, were kindly provided to us by The Senne Liquor. Although informed consent was not available due to the impossibility of contacting the patients in this retrospective study, the use of these samples was approved by the Ethics Committee of Universidade Santo Amaro, under protocol #55.272.749.

## 4 Results

Both MS and IIH groups presented similar age and sex distribution. CSF analysis revealed a slightly higher leukocyte count in MS, while the red blood cells (RBCs) count was higher in IIH. In the differential cell count, lymphocytes showed higher count in MS (91.7 ± 4.37 cells/mm^3^) than IIH (68.3 ± 37.64 cells/mm^3^) (Table 1).

**Table 1:** Age, sex, cytology, biochemistry, and OCBs analysis for CSF samples in both MS and IIH groups.

Descriptor	MS	IIH
Age (years) SD	34.8 ± 17.04	33.28 ± 14.41
Female	18	20
Male	7	5
*Citology analysis*		
Leukocytes (cells/mm^3^)	5.41 ± 6.23	1.2 ± 1.75
Red blood cells	31.71 ± 85.05	79.8 ± 383.4
*Biochemistry analysis*		
Total protein (mg/dL)	32.3 ± 10.91	29.8 ± 13.0
Glucose (mg/dL)	58.3 ± 20.58	58.3 ± 11.59
Lactate (mg/dL)	12.3 ± 4.45	13.38 ± 2.60
IgG index	1.0 ± 0.5	NA
*OCBs classification (%)*		
Type 2	20 (80%)	NA
Type 3	5 (20%)	
*OCBs count*		
2–7	9 (36%)	NA
> 8	16 (64%)	

Abbreviations: CSF, cerebrospinal fluid; IIH, idiopathic intracranial hypertension; MS, multiple sclerosis; NA, not applicable; OCBs, oligoclonal bands.

### 4.1 Anti‐HERV and Anti‐MOG Elisa Assay

Table 2 shows the synthesized peptide sequences (Peptide) from HERV‐W (1) and HERV‐K (2–6), and corresponding similarities between HERV and MOG sequences. There were at least eight sites of similarity between HERVs and MOG.

**Table 2:** Comparison between synthesized HERV peptides and MOG.

ID	Peptide	Peptide position	HERV	%; #aa	MOG position	Residues MOG
1‐W	LVGPLVSNL	1	LV**GPL**VS	57.1; 7	217	VL**GPLV**A
2‐K	PWEWGEKGISTP	9	I**S**T**P**	50.0; 4	4	L**S**R**P**
3‐K	WSGNQTLETRD	3	**G**N**Q**TL**E**T**R**	50.0; 9	88	**G**D**Q**AP**E**Y**R**
4‐K	LGIPTYAMS	4	**P**T**Y**AM**S**	50.0; 6	147	**P**F**Y**WV**S**
5‐K	ECVANSAVIL	7	**A**V**I**L	50.0 4	223	**A**L**I**I
		5	**P**S**F**G**R**	60.0 5	71	**P**P**F**S**R**
6‐K	VTHVPSFR	4	**VP**SF**G**	60.0; 5	215	**VP**VL**G**
		2	**T**HV**P**S**F**G**R**	50.0; 8	196	**T**FD**P**H**F**L**R**

*Note:* Bold indiates similar residues in the sequence of HERV and MOG. Percentage of similarity (%). Number of similar residues (#a, aa).

**Figure 1:**
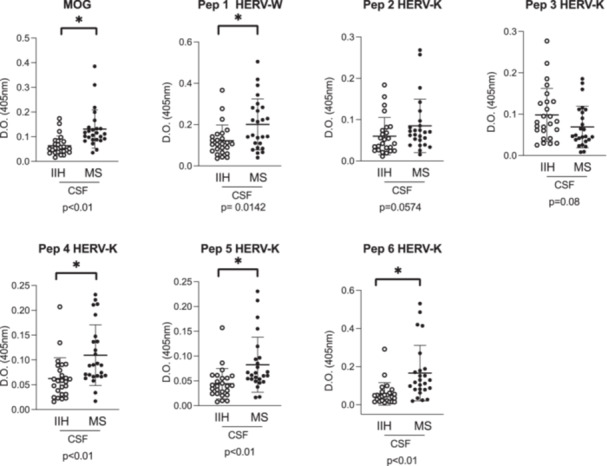
Anti‐MOG, anti‐HERV‐W, and anti‐HERV‐K humoral response in CSF of MS patients and IIH patients. MOG, HERV, and peptides 4, 5, and 6 from HERV‐K were statistically significantly higher in MS patients. Lines indicate the means and standard deviation. *p < 0.01.

MS patients presented significantly higher antibody concentrations to MOG (*p* < 0.01), HERV‐W (*p* = 0.0142) and the HERV‐K peptides 4 (*p* < 0.01), 5 (*p* < 0.01), and 6 (*p* < 0.01) when compared to IIH patients (Figure 1).

**Figure 2:**
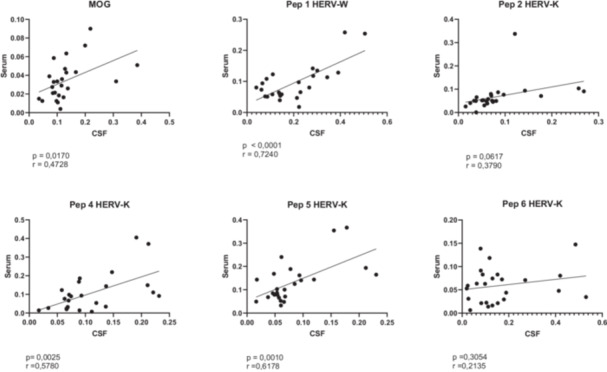
Pearson's coefficient correlation analysis indicates positive correlation between anti‐MOG antibody levels in CSF and serum, anti‐HERV‐W antibody levels in CSF and serum and anti‐HERV‐K antibody levels in CSF and serum (peptides 2, 4, and 5).

Next, we sought to determine whether anti‐HERVs and anti‐MOG antibodies in the serum were correlated with the antibody levels in the CSF. Pearson correlation test revealed a positive correlation to MOG, HERV‐W, and two HERV‐K peptides (4 and 5) between the antibody's levels in the CSF and serum (Figure 2).

Lastly, we have assessed whether anti‐MOG antibodies concentration in CSF and Serum in MS patients correlated with the concentration of the anti‐HERV‐K and HERV‐W peptide antibodies. In Table 3, we summarize the findings which showed statistical significance.

**Table 3:** Pearson's coefficient correlation analysis anti‐MOG antibodies concentration in the serum of MS patients and CSF with HERV‐W and HERV‐K peptides.

Anti‐MOG CSF			Anti‐MOG serum		
*x*	*r*	*p*	*x*	*r*	*p*
Pep 2 HERV‐K CSF	0.677	0.0002	Pep 1 HERV‐W CSF	0.578	0.002
Pep 2 HERV‐K serum	0.651	0.0004	Pep 2 HERV‐K CSF	0.745	< 0.0001
Pep 5 HERV‐K CSF	0.885	0.0001	Pep 2 HERV‐K Serum	0.847	< 0.0001
Pep 5 HERV‐K serum	0.596	0.0016	Pep 5 HERV‐K CSF	0.529	0.006
Pep 6 HERV‐K CSF	0.638	0.0006	Pep 5 HERV‐K Serum	0.809	< 0.001
			Pep 6 HERV‐K CSF	0.654	0.0004
			Pep 6 HERV‐K Serum	0.495	0.02

## 5 Discussion

Our findings indicated high concentrations of anti‐HERV‐W, anti‐HERV‐K, and anti‐MOG antibodies in the CSF of MS patients when compared to IIH patients, corroborating previous studies which described the presence of anti‐HERV‐W and K in CSF of MS patients [19, 20]. Our study focused on assessing the antibody response against HERV‐W and K peptides that showed similarity with MOG, one of the key proteins to the MS immunopathogenic response [21]. We have detected anti‐HERV antibodies directed to distinct sites of HERVs proteins and also distinct HERV families. Importantly, most evidence points to the role of HERV‐W in MS pathogenesis [6, 10, 22, 23, 24] and there is a lack of studies indicating association to other HERVs families to MS pathogenesis. More recently, transcriptome data from MS patients found 19 differentially expressed HERV families besides HERV‐W [4]. Taken together, these findings might indicate a diverse and complex response against distinct sites of HERVs proteins, and somehow the autoimmune response might be triggered by several sites of HERV that are similar to self‐antigens, and in other diseases that also present inflammatory response, HERV‐W is positively correlated to the inflammatory status [25]. In fact, all peptides designed for this study showed approximately 50% similarity to MOG, and therefore, may be potential candidates for autoimmune response triggered by molecular mimicry. Indeed, this first observation highlights the possible role of HERVs promoting complex inflammatory and autoimmune responses.

Interestingly, most studies have focused on studying HERV‐K gag or envelope proteins [19]. Here, we described the humoral response directed to the pol gene in the CSF and serum, which was not previously reported. Our findings might indicate that other genes may also be responsible for playing a role in MS pathogenesis.

Previous studies have also described the detection of antibodies against MOG in the CSF and serum of MS patients [26, 27, 28]. The detection of intrathecal anti‐MOG antibodies has been implied to be a potential biomarker of the disease and to presume the clinical status of MS [29]. Here, we described a positive correlation of anti‐MOG antibodies in serum and CSF, which indicates a systemic condition of anti‐MOG antibodies circulation/production. Also, we have detected positive correlation of anti‐MOG concentration in CSF and serum with many of the HERV peptides. These observations might indicate that these peptides could be possible target for autoimmune response specially through complex molecular mimicry. Other sites between MOG and HERV similarity was previously described [30, 31], which indicates that possibly many regions of the MOG might be targeted by anti‐HERV antibodies, and maybe this antibody mediated response could play a role in the MS pathogenesis against distinct sites MOG. Also, the concentration of antibodies against MOG positively correlates with the concentration of other anti‐HERV‐W and HERV‐K antibodies. We believe that these findings may pave the road to better understanding the role of HERVs in the MS pathogenesis. However, this observation should be tested in experimental model of MS to bring evidence to this hypothesis. In addition to this finding, the positive correlation between the concentration of anti‐MOG antibodies in the CSF and in the serum denotes that anti‐MOG serum quantification could be a less invasive approach with clinical relevance for MS prognosis. However, clinical trials should be performed to confirm this hypothesis.

Our study presents some limitations which include: (i) For IIH patients, we did not have access to serum samples, only CSF. Therefore, the comparison of the antibody levels in the serum between MS and IIH was not assessed, also we have not performed the differential expression analysis of HERVs in these samples. However, we observed a significantly high concentration of anti‐HERV‐K and ‐W antibodies in both serum and CSF of MS with a significant positive correlation, which indicates that the levels of anti‐HERV antibodies are high in both fluids. (ii) The samples were obtained retrospectively from a clinical laboratory which, by ethical considerations, does not collect the clinical data and related metadata from the patients. Thus, due to the lack of medical records of MS patients, we were not able to compare the anti‐HERV‐K, anti‐HERV‐W, and anti‐MOG concentration with the type of MS or expanded disability status scale (EDSS). However, we were able to observe intrathecal antibodies against HERV‐W and ‐K peptides, which brings some pieces to the MS pathogenesis puzzle. In fact, further studies should focus on investigating the level of anti‐HERV‐W and ‐K among the different types of MS and maybe further studies to determine the origin of these or other HERV transcripts to determine which loci could be potentially be similar and also expressed in the MS. (iii) Finally, we have selected restricted peptides which do not exclude other potential sites similar to MOG that might be of interest for triggering an autoimmune response. In this sense, other peptides should be considered to comprehensively understand the dynamics of humoral response against HERVs and self‐antigens.

## 6 Concluding Remarks

In summary, we have described the high antibody response against MOG‐like HERV‐W and ‐K peptides in the CSF. The findings described here may indicate a potential role of HERVs in autoimmune response through molecular mimicry. This mechanism could be triggered by a complex immune response against more than one site of MOG‐like proteins. Since several pieces of evidence corroborate the role of HERVs in MS pathogenesis and the concentration levels of anti‐HERV‐K, ‐W, and MOG antibodies of serum and CSF correlate positively, we believe that serum anti‐HERVs and anti‐MOG could be used as biomarkers for MS disease.

## Ethics Statement

This retrospective study was approved by the Ethics Committee of Universidade Santo Amaro, under protocol #55.272.749, the written consent was waived in order to the impossibility to reach out the patients. All collected samples were designated for diagnosis, and were not collected on purpose of answering the aim of this study, after the approve on the ethics committee the left‐over samples, that would be discarded, used on the study.

## Conflicts of Interest

The authors declare no conflicts of interest.

## Data Availability

The data generated and analyzed in the presented study are available upon request to the authors. The data are not publicly available due to privacy and ethical restrictions.
